# Acknowledgment to Reviewers of *Clocks & Sleep* in 2020

**DOI:** 10.3390/clockssleep3010010

**Published:** 2021-02-01

**Authors:** Clocks & Sleep Editorial Office

**Affiliations:** MDPI AG, St. Alban-Anlage 66, 4052 Basel, Switzerland

The peer review process represents the driving force of journal development, with reviewers acting as the gatekeepers who ensure that *Clocks & Sleep* maintains its high-quality standard of published papers. Thanks to the cooperation of our reviewers, in 2020, the median time to the first decision was 21 days and the median time to publication was 44 days. The editors would like to express their sincere gratitude to the following reviewers for their precious time and dedication, regardless of whether the papers were finally published:
Aarts, Mariëlle P. J.Martynowicz, HelenaAngerer, MonikaMatre, DagfinnAshton, AnnaMatsumoto, YukiBais, BabetteMayeuf-Louchart, AliciaBen Simon, EtiMcNeillis, SaraBender, Amy M.Miyata, SeikoBikov, AndrasMolle, MatthiasBonilla, Diego A.Murata, YusukeBorisenkov, M. F.Musiek, Erik S.Bracci, MassimoNagare, RohanBrown, Cary A.Natale, VincenzoCaetano, Gina MariaNgo, Hong-VietCordi, MarenO’Keeffe, KarynCori, JenniferPandey, Atul KumarDe Gennaro, LuigiPaulose, Jiffin K.Delaney, LoriPecor, KeithDumont, MariePeltz, JackFaraut, BricePérez-Fuentes, María Del CarmenFelder-Schmittbuhl, Marie-PaulePolak, AdamFernandez, Fabian-XoséPopkin, Stephen M.Figueiro, Mariana G.Postnova, SvetlanaFrau, RobertoPostuvan, VitaFrigato, ElenaRandjelovic, PavleGabryelska, AgataRasch, BjörnGarg, KawishRecchia, Daniela R.Georgian, BadicuReinke, HansGobrial, ErenyRiemma, GaetanoGordijn, MarijkeRinaldi, AlessandraGu, ZhenglinRoberts, SpencerGubin, DenisSandman, NilsHarbison, Susan T.Santamaria, JoanHarrison, Elizabeth M.Schmidt, ChristinaHonn, Kimberly A.Schreiner, ThomasHubbard, JeffreySilagadze, Zurab K.Jankowski, Konrad S.Spiegelhalder, KaiJawinski, PhilippeSprajcer, MadelineJin, JungheeSuarez-Garcia, AndresJoyce, DanielSulli, GabrieleKadotani, HiroshiSunde, ErlendKantermann, ThomasSyed, SheyumKompotis, KonstantinosSyed-Abdul, Majid MufaqamKooijman, SanderTaheri, PooyaLarson-Prior, Linda J.Vanderlinden, JulieLeach, JohnVetter, CélineLee, Li-AngViana, AlonçoLevent Sahin, LeventWakamura, TomokoLópez-López, DanielWalch, OliviaLuca, MariaWilson, SueMakarem, NourXiao, QianMaric, AngelinaZhang, ZhongxingMartiny, Klaus


